# Prevalence of new-onset migraine in patients with idiopathic intracranial hypertension in comparison to the general population

**Published:** 2018-10-07

**Authors:** Mansoureh Togha, Kamran Shirbache, Reza Rahmanzadeh, Zeinab Ghorbani, Zahra Yari, Farshid Refaeian, Shirin Behbahani, Parsa Panahi

**Affiliations:** 1 Iranian Center of Neurological Research, Neuroscience Institute, Tehran University of Medical Sciences, Tehran, Iran; 2 Sina Hospital, School of Medicine, Tehran University of Medical Sciences, Tehran, Iran; 3 School of Nutritional Sciences and Dietetics, School of Nutrition and Food Technology, Tehran University of Medical Sciences, Tehran, Iran; 4 Department of Clinical Nutrition and Dietetics, School of Nutrition and Food Technology, National Nutrition and Food Technology Research Institute, Shahid Beheshti University of Medical Sciences, Tehran, Iran

**Keywords:** Cerebrospinal Fluid, Headache, Idiopathic Intracranial Hypertension, Migraine, Papilloedema

## Abstract

**Background:** Idiopathic intracranial hypertension (IIH) encompasses patients with elevated intracranial pressure (ICP). Generally, it is difficult to make a differential diagnosis between IIH and co-existing migraine headaches. Thus, this article intends to estimate the prevalence of migraine in patients with IIH and explain the occurrence of new-onset migraine after the diagnosis of IIH.

**Methods:** The case group included 108 patients with IIH referred to the neurology wards of three university hospitals. A random sample of controls (n = 103) were recruited from patients hospitalized in the surgery and orthopedics ward. A checklist for migraine diagnosis was filled out. Cerebrospinal fluid (CSF) pressure and presence or absence of papilloedema (PE) in the patients and any necessary data were also recorded from the inpatient medical documents. All statistical analyses were done by SPSS software.

**Results:** There were 70 (64.80%) and 22 (21.40%) migraineurs in the case and control groups, respectively, and the difference was found to be significant (P < 0.001). In 26 (37.14%) migraine cases in the IIH group, the disorder was diagnosed after developing IIH. Also, there was a past medical history of having migraine in 44 (62.85%) migraineurs. In the fully adjusted regression models, the odds of being affected by migraine in patients with IIH was 6.17 times greater than the controls [odds ratio (OR) = 7.15, 95% confidence interval (CI) = 3.56-14.36, P < 0.010]. The patients’ mean CSF opening pressure was 32.10 ± 1.03 cmH_2_O and 93 (81.60%) subjects were found to have PE.

**Conclusion:** It was demonstrated that subjects with IIH might have about a 6-time higher likelihood of developing migraine headache than the general population. These considerations can help prevent misdiagnosis of migraine headache as the recurrence of IIH or uncontrolled IIH and subsequent inappropriate management.

## Introduction

According to the diagnostic criteria definition of International Headache Society (IHS), idiopathic intracranial hypertension (IIH) encompasses patients with elevated intracranial pressure (ICP) above 20 cmH_2_Oof unknown etiology with normal neuroimaging results.^1^^-^^5^ 

The onset of disease usually occurs in adulthood, between the ages of 11-58, and the mean age at the time of diagnosis is about 30 years.^6^^,^^7^ Although IIH should be considered as an important diagnosis due to allied morbidities, it is a relatively rare cause of headache with an annual incidence of 0.9 per 100000 in the total population, while the highest rate (3.5 per 100000) is seen among obese women of childbearing age.^7^^-^^9^ It has been reported that only around 10% of patients are men.^   10 ^

Headache is the most common symptom associated with IIH  ^11^^,^^12^ and the most concerning symptom is vision loss.^   6 ^ Papilloedema (PE) usually accompanies headache, both occurring in almost 90% of patients. The headache of IIH is mainly managed using medications that decrease ICP.^   13 ^ Other complications of IIH primarily include diplopia and tinnitus.^12^^,^^14^ In addition, migraine can coexist with IIH.^        15 ^ IIH is debated as a potential etiologic factor for migraine.^   16 ^ On the other hand, it has been shown that preexisting migraine may be exacerbated by IIH.^17^^,^^18^

Migraine is a pulsating and unilateral headache that can be accompanied by photophobia, phonophobia, and nausea.^   19 ^ Migraine is a major public health dilemma which adversely affects daily activities and can reduce quality of life.^   20 ^ This type of headache usually is accompanied by a number of comorbidities including gastrointestinal (GI) disorders and hypertension.   ^21^^,^^22^ As stated by the 2016 Global Burden of Disease (GBD), migraine is ranked as the first leading cause of disability in under 50 years, worldwide.^        23 ^ Studies have shown that about 10%-18% of general population is affected by migraine, with the susceptibility of women being three times more than men.^24^^,^^25^ The prevalence of migraine in the Iranian population has been estimated at 14%.^        26 ^

Generally, it is difficult to make a differential diagnosis between IIH and co-existing migraine headaches, unless other signs are present.^2^^,^^27^ On occasion, there are no traits that distinguish chronic daily headache due to IIH from chronic migraine.^   28 ^ MRI imaging criteria might be needed to prevent misdiagnosis of patients with IIH and distinguish them from those with chronic migraine headache.^        15 ^

Since apt and accurate diagnosis in a timely manner is crucial, especially when performing follow up on a patient’s status after IIH treatment, this article intends to estimate the prevalence of migraine in patients with IIH and explain the occurrence of new-onset migraine after the diagnosis of IIH.

## Materials and Methods

***Study subjects and data gathering:*** In this case-control study, the presence of migraine in patients with IIH was evaluated in comparison to non-IIH subjects. The diagnosis of IIH (with or without PE) and migraine was done according to the diagnostic criteria of increased lumbar puncture (LP) opening pressure in addition to ruling out all other probable conditions based on the International Classification of Headache Disorders-3^rd^ Edition (ICHD-3) (beta version) criteria.^   29 ^

The case group included 108 patients with IIH referred to the neurology ward of Sina, Shariati, and Imam Hossein university hospitals, Tehran, Iran. A random sample of controls (n = 103) were recruited from patients hospitalized in the surgery and orthopedics ward of Sina Hospital, Tehran City. The inclusion criteria comprised being under 60 years of age and IIH-diagnosed patients who were treated and cured of IIH (for the case group), and the exclusion criteria were presence of accompanying neurologic diseases (stroke, brain tumors, epilepsy, any type of headache other than migraine) and unwillingness to take part in the study. In order to collect the required information, all subjects were interviewed by a trained medical student. A checklist for migraine diagnosis was filled out. Demographic data were also collected. The subjects’ height and weight were recorded and the body mass index (BMI) was also determined. Cerebrospinal fluid (CSF) pressure and presence or absence of PE in the patients and any necessary data were also recorded from the inpatient medical documents.

The normal distribution of data was examined using the Kolmogorov-Smirnov test (K-S test). Then, Pearson’s chi-square test and the Student’s t-test or the Mann-Whitney U test (as appropriate) were used for comparing the statistical difference between the categorical and continuous variables, respectively. Descriptive data were presented using tables and graphs. Unadjusted and adjusted logistic regression models were applied to analyze the association between the prevalence of migraine and IIH. The corresponding odds ratios (ORs) and their associated confidence intervals (95% CI) were determined. P-values less than 0.050 were considered to be statistically significant. All statistical analyses were done by SPSS software (version 19, SPSS Inc., Chicago, IL, USA).

## Results

**Figure 1:**
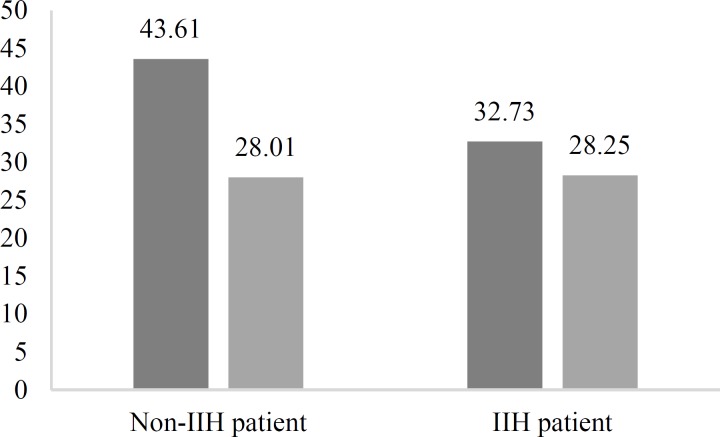
Age and body mass index (BMI) of studied population

In the present study, 211 subjects (28 men, 13.3%; 183 women, 86.7%) consisting of 108 patients with IIH (20 men, 18.5%; 88 women, 81.5%) and 103 non-IIH control individuals (8 men, 7.8%; 95 women, 92.2%) were enrolled. Using the independent sample t-test, the mean BMI of the case group (28.00 ± 4.62) was comparable to that of the control group (28.25 ± 5.26). Age had a statistically significant difference between cases (32.73 ± 10.32) compared to controls (43.61 ± 13.16) (P < 0.001) (Figure 1).

IIH: Idiopathic intracranial hypertension

The prevalence of migraine between the 2 groups was compared. There were 70 (64.80%) and 22 (21.40%) migraineurs in the case and control groups, respectively, and the difference was found to be significant (P < 0.001). The time of migraine diagnosis in patients with IIH is summarized in table 1. 

**Table 1:** Time of migraine diagnosis in patients with idiopathic intracranial hypertension (IIH**)**

**Group of patients**	**Number of ** **migraineurs (%)**
Past medical history of migraine (before IIH diagnosis)	44 (62.85)
New-onset migraine (after IIH diagnosis)	26 (37.14)
Migraine relief after treating IIH	12 (17.14)

IIH: Idiopathic intracranial hypertension

**Figure 2:**
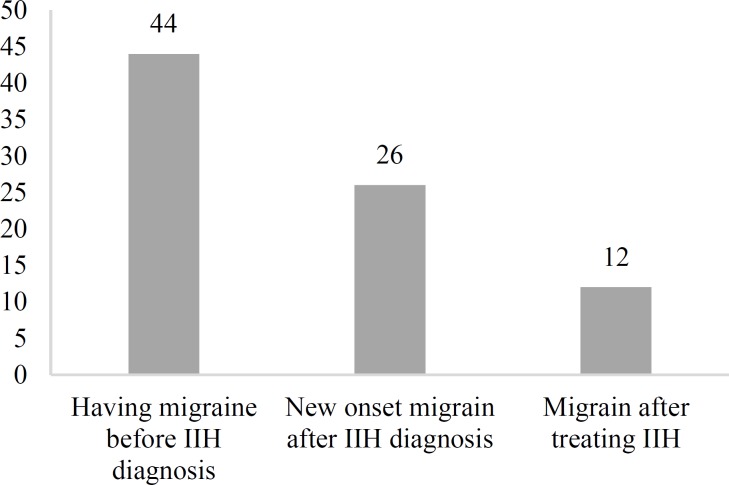
Time of migraine diagnosis in patients with idiopathic intracranial hypertension (IIH)

In 26 of 70 (37.14%) migraine cases in the IIH group, the disorder was diagnosed after developing IIH and the patients did not have any symptoms of migraine before IIH diagnosis. There was a past medical history of having migraine in 44 of 70 (62.85%) migraineurs in the IIH group. Migraine was relieved in 12 patients (17.14%) after the treatment of IIH (Figure 2).

Thus, our results demonstrated that patients with IIH had approximately 7 times the odds of having migraine compared to non-IIH individuals (OR = 6.78, 95% CI = 3.66-12.54, P < 0.010). After adjusting the regression models for age and BMI, the odds of being affected by migraine in patients with IIH was 6.17 times greater than the controls (OR = 7.15, 95% CI = 3.56-14.36, P < 0.010).

Patients with IIH had undergone LP to evaluate CSF opening pressure and were also examined for PE. The patients’ mean CSF opening pressure was 32.10 ± 1.03 cmH_2_O (range: 21-65 cmH_2_O) and 93 (81.60%) subjects were found to have PE. Comparison of CSF pressure in PE and non-PE subjects, showed that mean CSF pressure was significantly higher in PE cases (33.06 cmH_2_O) than non-PE cases (26.13 cmH_2_O) (P = 0.004); however, when comparing the CSF pressure between patients with IIH with or without migraine, the difference was no longer significant (Table 2).

**Figure 3:**
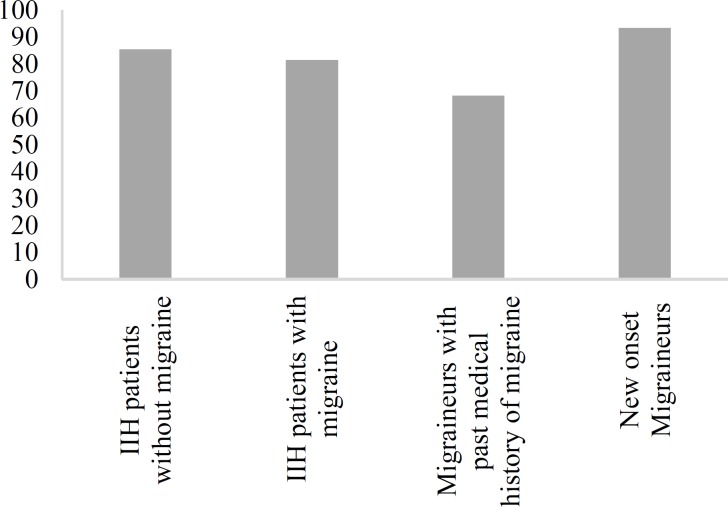
Prevalence of papilloedema (PE) among subgroups of patients with idiopathic intracranial hypertension (IIH)

Presence of PE was further explored in the subgroups of patients with IIH. PE prevalence was 85.7% (60 of 70 patients) and 86.8% (33 of 38 patients) in patients with and without migraine, respectively. Also, 79.5% (35 of 44 patients) of migraineurs with past medical history of migraine and 96.1% (25 of 26 patients) of new-onset migraineurs had PE (Figure 3).

**Table 2:** Comparison of cerebrospinal fluid (CSF) pressure in different subsets of patients with idiopathic intracranial hypertension (IIH)

**Group of patients**	**Mean ± SD**	**Number of patients**	**P**
All patients with IIH	32.10 ± 1.03	108	
Patients with IIH not having PE	26.13 ± 2.82	15	- 0.004
Patients with IIH having PE	33.06 ± 10.84	93	
Patients with IIH not having migraine	32.84 ± 11.84	38	0.196
Patients with IIH having migraine	31.70 ± 9.57	70	
Past medical history of migraine (before IIH diagnosis)	31.40 ± 10.04	82	
New-onset migraine (after IIH diagnosis)	34.28 ± 11.31	26	

IIH: Idiopathic intracranial hypertension; PE: Papilloedema; SD: Standard deviation

## Discussion

Based on our results, the odds of having migraine in patients with IIH is approximately 7 times higher than that in non-IIH individuals, which is in line with the findings of Mathew, et al.^   28 ^ Their observations indicated a link between migraine and IIH; however, they stated that further studies were required to confirm their findings.

In our study, the prevalence of migraine in patients with IIH was 64.80%, of which migraine was relieved in 17.14% of patients. Moreover, the prevalence of new-onset migraine after diagnosis and initiation of treatment of IIH was estimated at about 37.14%, while around 62.85% of the migraineurs had a past medical history of migraine. These results further support the findings obtained by Friedman and Rausch who showed that among 82 patients with IIH (80 women, aged 10-59 years), 11 patients (13.41%) had a previous history of headache and 56 subjects (68.0%) developed headache after applying treatment options for IIH, of which 20.0% had migraine without urea; thus, the researchers concluded that due to the possibility of different types of headache in patients with IIH, taking a precise history and performing a detailed clinical examination was vital for making a proper diagnosis and administration of the appropriate treatment.^   2 ^ In another study conducted by Sina, et al. on 68 patients with IIH in Iran, it was reported that 63.4% of individuals suffering from IIH had a past medical history of migraine, of which 82.2% were female patients.^   30 ^ In addition, another study showed that the prevalence of headache among 40 patients with IIH was about 75% of which 43.3% had some symptoms attributed to migraine.^   31 ^

As it has been confirmed by extensive evidence, overweight and obesity play a distinct role in predisposing an individual to developing IIH with higher probability compared to a non-obese subject. Also, it has been shown that losing weight in patients with IIH can be an effective treatment strategy for the disorder.^32^^,^^33^ On the other hand, it has been reported that obesity may be a risk factor for migraine, and developing therapeutic strategies and utilization of weight loss techniques can result in improving migraine symptoms.^22^^,^^34^^,^^35^ Thus, we considered obesity as one of the important confounders for evaluating the association between migraine and IIH. It was demonstrated that, when considering the age and BMI in fully adjusted regression models, the odds of being affected by migraine in patients with IIH was 6.17 times greater than the controls (OR = 7.15, 95% CI = 3.56-14.36, P < 0.001). 

One of the suggested mechanisms for the association between increased ICP and augmented pain in migraine might be related to trigeminovascular firing at the level of complete sinus stenosis.^   36 ^

The relationship between high CSF pressure and migraine is still a subject of discussion. Van Alphen examined 40 patients and concluded that increased CSF pressure could account for headache and the neurologic manifestation of migraine.^   37 ^ However, our results comparing CSF pressure between patients with IIH with and without migraine showed no significant difference. Another study also stated that CSF pressure had no correlation with the degree of improvement in antimigraine and diuretic therapy.^   28 ^

Headache is the most common nonvisual complaint of IIH and PE is seen in more than 90% of patients with IIH.^   38 ^ In the present study, 93 (81.6%) subjects among the patients with IIH were found to have PE. This result is in consonance with previous reports.^39^^-^^41^

Torbey, et al. examined the efficacy of CSF pressure assessment in order to diagnose IIH without PE in patients with chronic daily headache. The mean CSF pressure of the IIH cases was reported at 30 cmH_2_O and the minimum pressure was recorded when patients were asleep (10-14 cmH_2_O).^   42 ^ Also, D’Amico, et al. reported the mean CSF pressure of patients with IIH at 29.79 cmH_2_O in their research.^   3 3^ In our study, the mean CSF pressure of the patients was 31.10 cmH_2_O, which is close to that reported in these studies. 

In addition, our findings showed that mean CSF pressure was significantly higher in PE cases than non-PE patients (33.06 vs. 26.13 cmH_2_O, P < 0.010). These results are in line with the results of a cross-sectional analysis of 353 patients with IIH for whom mean opening pressures were lower for those without PE compared to those with PE (30.90 vs. 37.30 cmH_2_O).^   41 ^

In another analysis, we compared the CSF pressure among patients with IIH with and without migraine. However, our findings demonstrated that the mean CSF pressure of patients with IIH with and without migraine was similar. We also did not witness any significant difference in the BMI of the studied groups. These results differ from those reported by Vieira, et al. in which it was described that 10% of 62 patients with chronic migraine raised CSF pressure and obesity was a risk factor for developing IIH and suggested LP for all obese migraineurs.^   43 ^

The strengths of the present study were the use of standard diagnostic criteria for both IIH and migraine and including a control group which represented the general population.

However, this study also has a number of limitations such as the retro-prospective nature and the lack of CSF pressures for normal subjects which were ethically and logically impossible to obtain.

## Conclusion

Due to the higher prevalence of migraine and even the possibility of developing new-onset migraine in patients with IIH, taking precise headache histories and preforming exact examinations are crucial. It was demonstrated that subjects with IIH may have about a 6-time higher likelihood of developing migraine headache than the general population. These considerations can help prevent misdiagnosis of migraine headache as the recurrence of IIH or uncontrolled IIH and subsequent inappropriate management. On the other hand, IIH may be presented without PE and might be misdiagnosed as chronic migraine. Therefore, a two-way approach to patients with chronic headache is necessary.

## Notes:

**How to cite this article:** Togha M, Shirbache K, Rahmanzadeh R, Ghorbani Z, Yari Z, Refaeian F, et al. Prevalence of new-onset migraine in patients with idiopathic intracranial hypertension in comparison to the general population. Iran J Neurol 2018; 17(4): 161-6.
